# Quantitative proteomics reveals molecular mechanism of gamabufotalin and its potential inhibition on Hsp90 in lung cancer

**DOI:** 10.18632/oncotarget.10388

**Published:** 2016-07-02

**Authors:** Liyuan Zhang, Zhenlong Yu, Yan Wang, Xiaobo Wang, Lianru Zhang, Chao Wang, Qingxi Yue, Xun Wang, Sa Deng, Xiaokui Huo, Xiangge Tian, Shanshan Huang, Baojing Zhang, Xiaochi Ma

**Affiliations:** ^1^ Dalian Medical University, Dalian, China; ^2^ Department of Pharmacy and Traditional Chinese Medicine, Chinese People's Liberation Army 210 Hospital, Dalian, China; ^3^ State Key Lab of Cellular Stress Biology, School of Life Sciences, Xiamen University, Xiamen, China; ^4^ Institute of Oncology, Shanghai 9th People's Hospital, Shanghai JiaoTong University School of Medicine, Shanghai, China; ^5^ Department of Nuerosurgery, The Third People's Hospital of Dalian, Non-Directly Affiliated Hospital of Dalian Medical University, Dalian, China

**Keywords:** gamabufotalin, heat shock protein 90 (Hsp90), quantitative proteomics, gene ontology (GO), NSCLC

## Abstract

Gamabufotalin (CS-6) is a major bufadienolide of *Chansu*, which shows desirable metabolic stability and less adverse effect in cancer therapy. CS-6 treatment inhibited the proliferation of NSCLC in a nanomolar range. And CS-6 could induce G2/M cell cycle arrest and apoptosis in A549 cells. However, its molecular mechanism in antitumor activity remains poorly understood. We employed a quantitative proteomics approach to identify the potential cellular targets of CS-6, and found 38 possible target-related proteins. Among them, 31 proteins were closely related in the protein-protein interaction network. One of the regulatory nodes in key pathways was occupied by Hsp90. Molecular docking revealed that CS-6 interacted with the ATP-binding sites of Hsp90. In addition, CS-6 inhibited the chaperone function of Hsp90 and reduced expression of Hsp90-dependent client proteins. Moreover, CS-6 markedly down-regulated the protein level of Hsp90 in tumor tissues of the *xenograft* mice. Taken together, our results suggest that CS-6 might be a novel inhibitor of Hsp90, and the possible network associated with CS-6 target-related proteins was constructed, which provided experimental evidence for the preclinical value of using CS-6 as an effective antitumor agent in treatment of NSCLC.

## INTRODUCTION

Lung cancer remains the leading cancer killer around the world [1], especially in China [2], over 85% of lung cancers are diagnosed as non-small cell lung cancer (NSCLC), with 1-year survival rate below 15% [3]. Advanced NSCLC is often treated with radiation therapy or chemotherapy, which could lead many side effects and even increase a person's risk in developing second cancers later in life. More research is urgently needed to explore new therapy strategies that not only improve the survival rate, but also eliminate suffering of patients with lung cancer.

Most recently, natural products isolated from traditional Chinese medicines have drawn more attentions, due to their great potential in antitumor activities [4, 5] with low toxicity and few side effects. Bufadienolides are the major bioactive constituents found in Toad venom, which is from the postauricular glands and skin secretions of *Bufo bufo gargarizans* Cantor or *Bufo melanostictus* Schneider [6]. Several *in vitro* and *in vivo* studies have demonstrated anti-cancer effects of bufadienolides in various cancers [7, 8]. Gamabufotalin (CS-6), a major derivative of bufadienolides, has shown significant anti-tumor activity, with stable metabolic properties and less adverse effects in previous work [9–11], compared with other bufadienolides. However, as a bioactive molecular, the key signal pathways underlying the anti-cancer mechanism, and the therapeutic targets of CS-6 had not yet been well characterized.

Quantitative proteomics techniques have recently emerged as a robust tool to uncover the differential proteins expression associated with mechanism and therapeutic targets of natural products. The most accurate mass spectrometry based quantitative approach is typically performed by stable isotope labeling. Stable isotope dimethyl labeling [12–14] provides a very straightforward, fast and inexpensive means for quantitative proteomics, compared with other quantitative strategies.

In the present study, the molecular mechanism of the anti-cancer effects of CS-6 was deciphered by interrogating the proteomics changes incurred in A549 cells after exposure to CS-6. For this purpose, stable isotope-based dimethyl labeling together with nanoscale liquid chromatography-mass spectrometry were employed. The quantitative proteomics combined with bioinformatics has been proven to be a powerful tool to reveal the complex molecular events in biological systems, which enable us to quantitate the changes in proteins from a broad array of biochemical and signaling pathways. Thus, a comprehensive bioinformatics analysis was conducted to figure the biological pathways in response to CS-6 activation. The results indicated that several key proteins in cell signaling and tumor regeneration, including heat shock protein 90 (Hsp90), might be involved in the anti-cancer effect of CS-6 in A549 cells. The predicted binding between CS-6 and Hsp90 was then verified by molecular docking. Our results show that CS-6 might be a potential inhibitor of Hsp90, which shed more light on the mechanism of anti-cancer effects of CS-6.

## RESULTS

### Effects of CS-6 on cell viability and morphological changes of A549 cells

**Figure 1:**
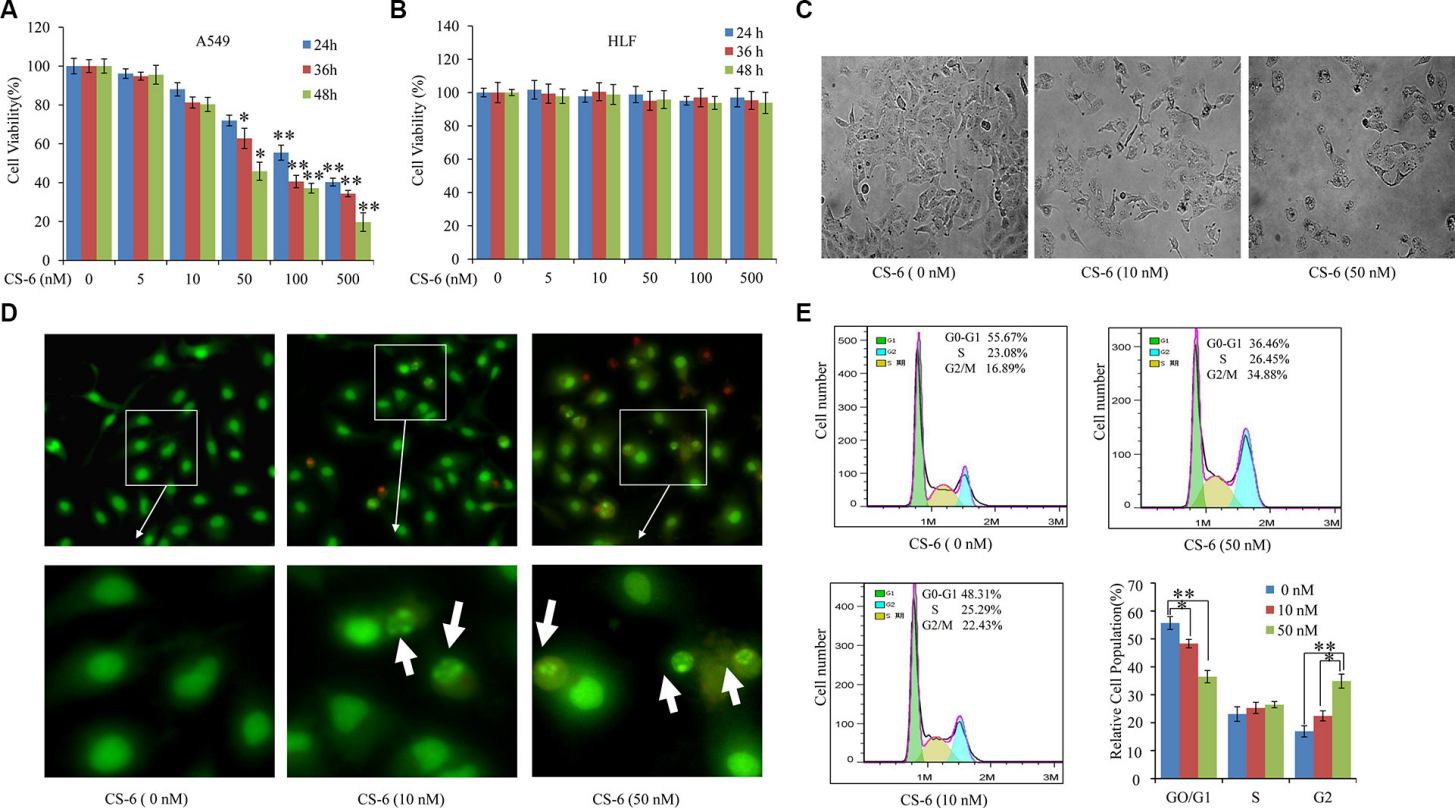
CS-6 inhibited cell viability and changed morphology (**A**) A549 and (**B**) HLF cells were treated with 5, 10, 50, 100 and 500 nM CS-6 for 24, 36 and 48 h, and cell viability was determined by MTT assay. (**C**) Morphological change induced by 10 and 50 nM CS-6 in A549 cells after 48 h treatment (200×, magnification). Typical apoptotic morphological change in CS-6-treated cells was observed. The data are presented as mean ± SD of three tests. (*p < 0.05, **p < 0.01, significant differences between CS-6 treatment groups and DMSO vehicle control groups). (**D**) Fluorescence images of A549 cells stained by AO/EB (100×, magnification), and nuclear apoptotic bodies (weight arrow) were formed in CS-6-treatet groups. (**E**) Cell cycle arrest of the cells induced by CS-6. Data are presented as mean ± SD (*n* = 3). *p < 0.01, **p < 0.01 compared with control group.

As shown in Figure 1A, the survival rate of A549 cells was reduced in a dose- and time-dependent manner after treatment with increasing concentrations of CS-6 (0, 5, 10, 50, 100 and 500 nM) for 24, 36 and 48 h. However, no obvious cytotoxicity was observed in human normal lung cell line (HLF cells) at the same dose (Figure 1B). The IC_50_ value of CS-6 was 48.4 ± 2.5 nM for 48 h treatment. The morphological changes of A549 cells caused by 36 h exposure to the various concentrations of CS-6 (0, 10 and 50 nM) are shown in Figure 1C. As the concentration of CS-6 increased, shrunk cells and plasma membrane blebs were exhibited. The AO/EB double staining showed that viable cells with intact DNA and nucleus show a round and green nuclei, while late apoptotic and necrotic cells with fragmented DNA display an orange to red nuclei. As shown in Figure 1D, CS-6 exposure induced late apoptotic and necrotic cells, and increasing concentration of CS-6, the number of viable cells decreased tremendously. In addition, the chromatin of nuclei condensed and nuclear apoptotic bodies were formed when cancer cells were treated with CS-6. Furthermore, as shown in Figure 1E, after treatment with CS-6 for 36 h, the representative DNA histograms of A549 cells showed that the percentages of G0/G1 phase were decreased from 55.67% to 36.46%, whereas G2/M phase were increased from 16.89% to 34.88%, S phase were not affected by 50 nM CS-6, compared with the control group. These results fully indicated that CS-6 at 10 and 50 nM could both induce G2/M phase arrest of A549 cells. Together, these results suggested that CS-6 could trigger the programmed cell death of A549 cells, while it have no obvious impact on the normal cells.

### Effect of CS-6 on proteome profile of A549 cells

Accurate and high throughput quantification of differentially expressed proteins is great essential to further decipher the mechanism of cell toxicity induced by CS-6; stable isotope dimethyl labeling based quantitative proteomics strategy with the advantages of low cost, rapid and high derivatization efficiency was employed. As a result, 493 proteins were quantified in all thrice quantification when controlling the RSD of the ratios for the quantified proteins to less than 50%, of which, 38 proteins were quantified with at least a 2-fold change with statistical significance (*p*-value < 0.05) (Table 1).

**Table 1:** Details about differentially expressed proteins upon CS-6 treatment, showing > 2 fold up- or down-regulation with statistical significance

No.	IPI Accession	Gi Accession	Protein Name	Pepties matched	Unique peptides	Sequence Coverage (%)	MW	Ratio of D to H	*p*-value
1	IPI00014230	C1QBP	Complement component 1 Q subcomponent-binding protein	6	6	32.6	31.362	0.35	0.001
2	IPI00414676	HSP90AB1	Heat shock protein HSP 90-beta	30	9	43.9	83.263	0.41	0.005
3	IPI00024993	ECHS1	Enoyl-CoA hydratase, mitochondrial	2	2	12.1	31.387	0.41	0.016
4	IPI00023860	NAP1L1	Nucleosome assembly protein 1-like 1	6	5	25.1	45.374	0.32	0.003
5	IPI00026271	RPS14	40S ribosomal protein S14	1	2	15.1	16.273	0.31	0.017
6	IPI00293276	MIF	Macrophage migration inhibitory factor	1	1	7.8	12.476	0.28	< 0.001
7	IPI00026546	PAFAH1B2	Platelet-activating factor acetylhydrolase IB subunit beta	1	1	3.9	25.569	0.27	0.02
8	IPI00306332	RPL24	60S ribosomal protein L24	2	2	13.4	17.779	0.33	0.035
9	IPI00007797	FABP5	Fatty acid-binding protein, epidermal	2	2	22.2	15.164	0.30	0.008
10	IPI00789101	PTGES3	Prostaglandin E Synthase 3	4	4	30.3	19.448	0.32	0.005
11	IPI00219153	RPL22	60S ribosomal protein L22	3	3	39.1	14.787	0.31	0.031
12	IPI00646304	PPIB	Peptidyl-prolyl cis-trans isomerase B	9	8	38	23.742	0.30	0.005
13	IPI00013068	EIF3E	Eukaryotic translation initiation factor 3 subunit E	3	3	10.6	52.22	0.41	0.034
14	IPI00980853	RHOC	Rho-related GTP-binding protein RhoC	2	1	18.8	25.485	0.45	0.048
15	IPI00789041	PNN	Pinin	1	1	1.8	81.613	50.00	< 0.001
16	IPI00006167	PPM1G	Protein phosphatase 1G	1	1	5.3	59.271	0.17	< 0.001
17	IPI00761160	CAST	Calpastatin	1	1	3.7	84.942	0.34	0.017
18	IPI00013895	S100A11	S100 calcium binding protein A11	4	4	42.9	11.74	0.34	0.039
19	IPI00003217	PSMB7	Proteasome subunit beta type-7	4	4	23.8	29.965	0.41	0.044
20	IPI00219038	H3F3A	H3 Histone, Family 3A (H3F3A)	4	4	25	15.328	2.70	< 0.001
21	IPI00015361	PFDN5	Prefoldin subunit 5	1	1	11.7	17.328	0.29	< 0.001
22	IPI00013004	PDXK	Pyridoxal kinase	2	2	11.9	35.102	0.43	0.002
23	IPI00719622	RPS28	40S ribosomal protein S28	2	2	30.4	7.8409	0.37	0.004
24	IPI00219037	H2AFX	Histone H2AX	4	1	32.2	15.144	0.36	0.002
25	IPI00015077	EIF1	Eukaryotic translation initiation factor 1	3	3	45.1	12.732	0.35	0.009
26	IPI00165393	ANP32E	Acidic leucine-rich nuclear phosphoprotein 32 family member E	3	3	17.9	30.692	0.45	0.01
27	IPI00298547	PARK7	Parkinson disease protein 7	3	3	31.7	19.891	0.35	0.015
28	IPI00031420	UGDH	UDP-glucose 6-dehydrogenase	17	17	50.4	55.023	0.39	0.016
29	IPI00215914	ARF1	ADP-ribosylation factor 1	3	3	19.9	20.697	0.46	0.032
30	IPI00302850	SNRPD1	Small nuclear ribonucleoprotein Sm D1	1	1	16.8	13.281	0.37	< 0.001
31	IPI00021258	ARFIP1	ADP-ribosylation factor-interacting protein 1	1	1	2.7	41.738	8.33	< 0.001
32	IPI00031691	RPL9	60S ribosomal protein L9	4	4	36.5	21.863	0.41	0.026
33	IPI00176903	PTRF	Polymerase I and transcript release factor	1	1	4.6	43.476	0.38	0.004
34	IPI00376005	EIF5A	Eukaryotic translation initiation factor 5A-1	10	10	57.6	20.17	0.48	0.026
35	IPI00473079	UGT1A3	UDP-glucuronosy ltransferase 1A3	1	1	3.9	60.338	0.24	0.025
36	IPI00783982	COPG	Coatomer subunit gamma-1	2	2	2.9	97.717	0.40	0.039
37	IPI00791020	GALNT13	Polypeptide N-acetylgalactosam inyltransferase 13	1	1	1.1	64.624	27.80	< 0.001
38	IPI00005981	TAGLN3	Transgelin-3	1	1	6.3	25.01	7.76	0.001

**Figure 2:**
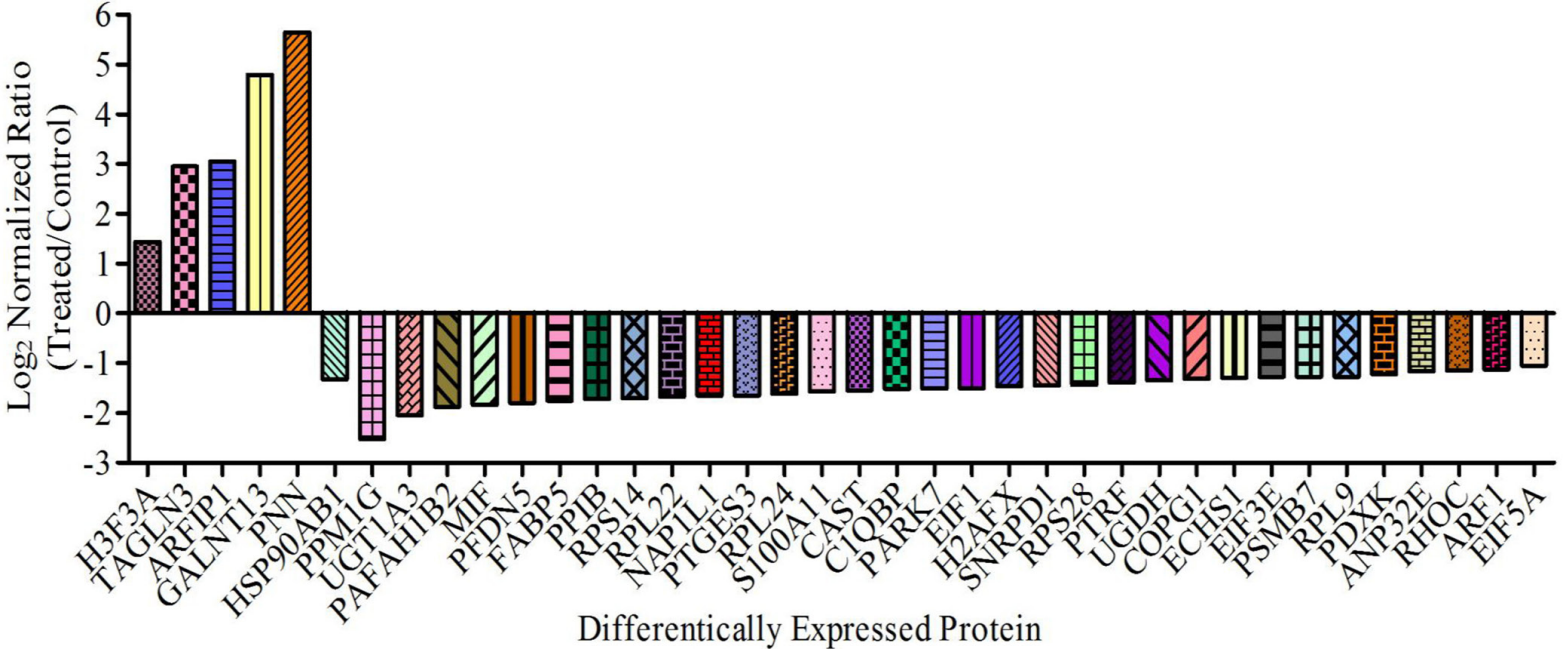
Proteins showing > 2-fold change in abundance with CS-6 treatment (95% confidence interval and *p*-value < 0.05) are shown

More detailed information about these differentially expressed proteins is presented in Table 1. Among these 38 differential proteins, 5 proteins were up-regulated (ratio > 2) and 33 proteins were down-regulated (ratio < 0.5) by the treatment of CS-6. The fold change and direction of CS-6-modulated proteins are represented in Figure 2.

### Gene ontology analysis of differentially expressed proteins

**Figure 3:**
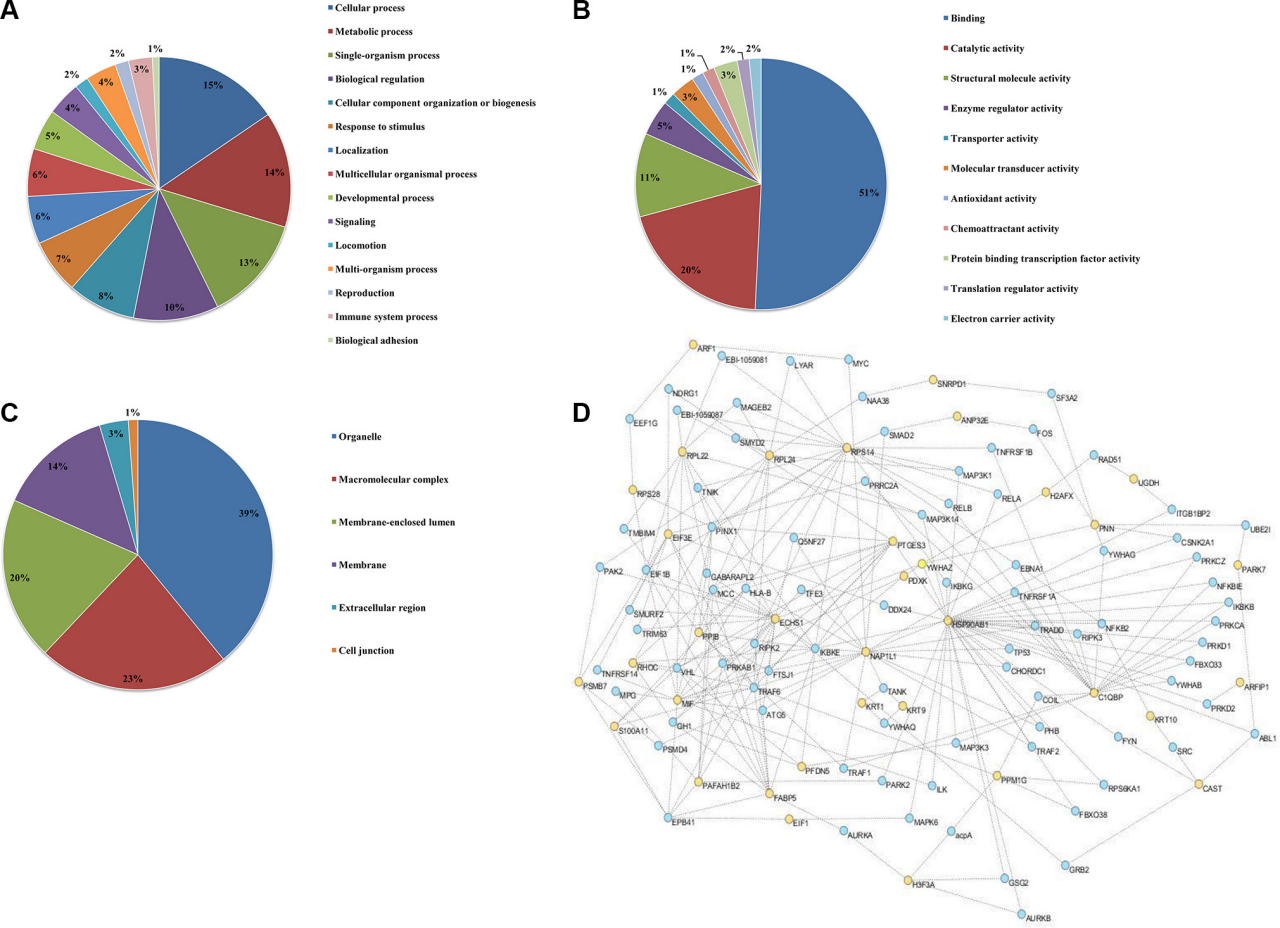
Gene ontology annotations of the proteome changes Identified proteins showing > 2 fold change were systematized on the basis of (**A**) biological processes, (**B**) molecular functions and (**C**) cellular component, using Blast2GO annotation. (**D**) Cytoscape 3.1 analysis shows the interaction network of the 31 differentially expressed proteins.

To better characterize the 38 differentially expressed proteins, the Gene Ontology (GO), including “Biological Processes”, “Molecular Function”, and “Cellular Component” was performed with Blast2GO using standard parameters. The biological processes of the significantly differentially expressed proteins were most enriched in regulation of cellular, metabolic, single-organism and biological process (Figure 3A). The categorical analysis of molecular function revealed that the majority of these proteins were classified into binding, catalytic activity, and structural molecule activity, and enzyme regulator activity (Figure 3B). Most of identified annotated proteins were located in the organelle, macromolecular complex (14%), membrane and membrane-enclosed lumen, respectively (Figure 3C). Taken together, the data from gene ontology reveal that CS-6 affects multiple critical cellular processes relevant to cell growth and proliferation.

Additionally, the protein-protein networks of CS-6 modulated proteins were analyzed with Cytoscape 3.1 network analysis. The networks formed by the identified proteins provided insights into the potential mechanisms and biological processes that regulated by CS-6. In this study, 31 proteins could link together into one network through direct interaction or only one intermediate partner (Figure 3D), suggesting the inherent correlation among all of them. These interacting proteins include the ribosome proteins, RNA transport proteins, apoptotic proteins and metabolic process proteins. It is interesting to note that the transcription factor NF-κB, was also observed in the network, which was reported as the potential target of CS-6 in our previous work [9].

### Confirmation of differentially expressed proteins by Western blotting

**Figure 4:**
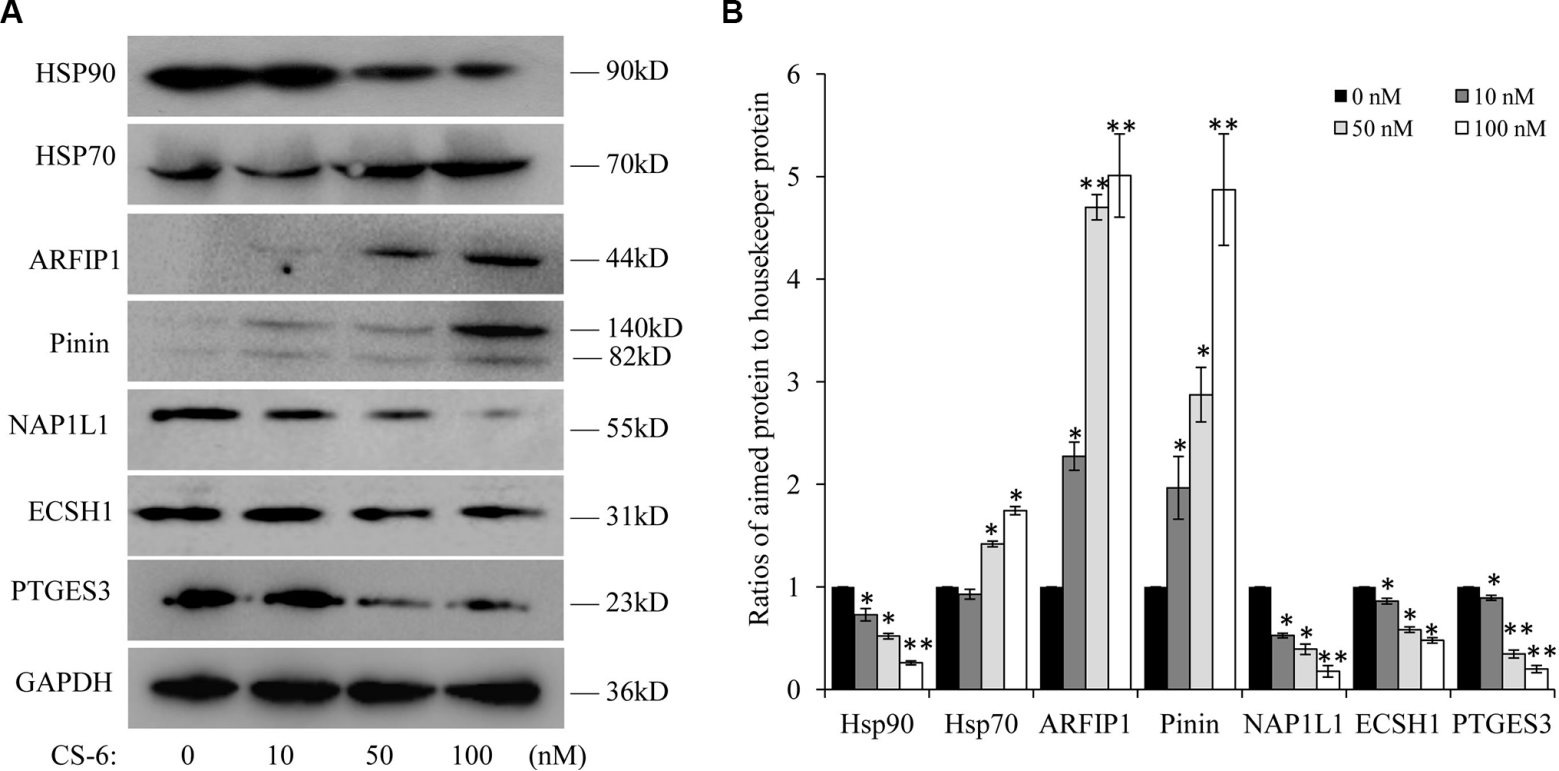
(**A**) Western blotting of Hsp90, Hsp70, ARFIP1, PNN, NAP1L1, ECSH1 and PTGES3, after treatment for 36 h. Each blot is the representative result of three independent experiments. (**B**) Quantitative data of (A). *p < 0.01, **p < 0.01 compared with control group.

Data from the mass spectrometric results and the protein-protein networks profiling shown by Cytoscape analysis have revealed the proteins differentially up and down regulated by CS-6. Many differentially expressed proteins are identified as multiple central nodes, such as Hsp90AB1 (Heat shock protein Hsp 90-beta), ECSH1 (Enoyl-CoA hydratase, mitochondrial), NAP1L1 (Nucleosome assembly protein 1-like 1), MIF (Macrophage migration inhibitory factor), PTGES3 (Prostaglandin E Synthase 3), PNN (Pinin) and *et al*. The expression of some identified target proteins were chosen to be validated in control and CS-6 treated A549 cells (for 36h) through western blotting assay. Consistent with the proteomics results, Hsp90, NAP1L1, ECSH1 and PTGES3 were found to be down-regulated, whereas ARFIP1 and PNN were found to be up-regulated in CS-6 treated A549 cells (Figure 4).

### Western-blotting analysis of senescence and growth associated Hsp90 client proteins after CS-6 treatment

**Figure 5:**
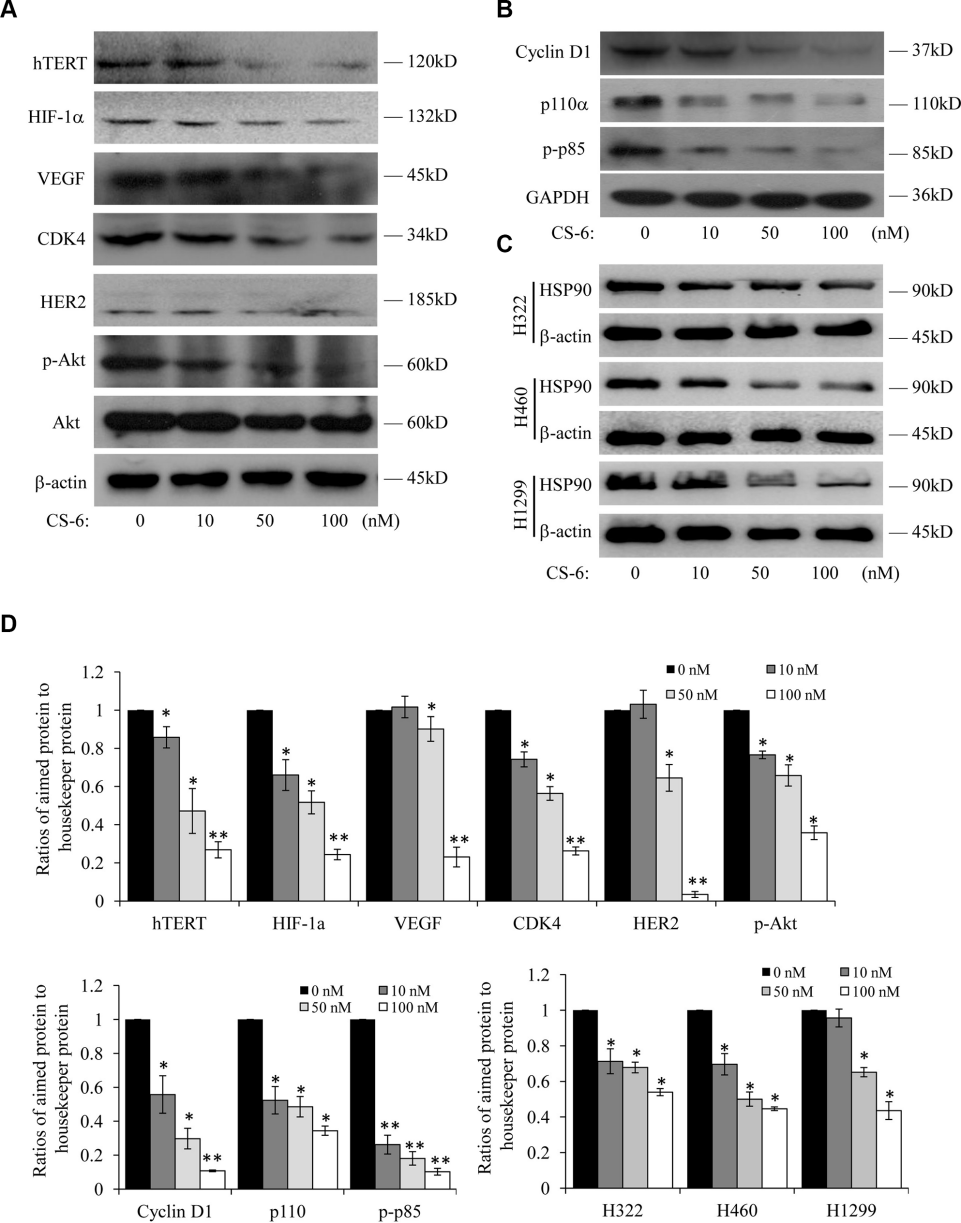
Western-blotting analysis of Hsp90 client proteins in CS-6-treated A549 cells (**A**) Expression of senescence and cell cycle associated Hsp90 client proteins. (**B**) Expression of associated Hsp90 client proteins. Each blot is the representative result of three independent experiments. (**C**) At 36 h after treatment, the Hsp90 protein levels were analyzed by Western blotting in H1299, H322 and H460 cells. β-actin was used as controls for sample loading. (**D**) Quantitative data of (A, B and C). *p < 0.01, **p < 0.01 compared with control group.

From the protein-protein networks, we found that Hsp90AB1 was the key central node, as it had the biggest interaction with other proteins in the network. In addition, our previous work demonstrated that CS-6 targeted IKKβ/NF-κB to prohibit lung cancer growth; however, IKK was one member of Hsp90 client protein [15], which also suggesting that Hsp90 was an important protein. We hypothesized that Hsp90AB1 was the most important protein molecules that was affected by CS-6 treatment in A549cells. Hsp90 is a molecular chaperone that plays an indispensable role in normal cellular homeostasis by regulating the folding, stability, and function of its target substrates, termed “client” proteins [16, 17], many of which have effects in regulating signal transduction pathways. We next detected the expression of Hsp90 client proteins after CS-6 treatment. The expression of senescence, proliferation and cell cycle associated Hsp90 client oncoproteins, like hTERT, HIF-1α, VEGF, CDK4, HER2 and Akt were analyzed in CS-6-treated A549 cells. At the concentration of 50 nM, the protein expression levels of hTERT, HIF-1α, VEGF, CDK4, HER2 and p-Akt were significantly reduced (Figure 5A), while Akt protein level didn't change obviously. Besides, the expression of some commonly overexpressed oncoproteins in NSCLC, known as proteins in Hsp90 client signaling pathway were also detected. As shown in Figure 5B, the expression of Cyclin D1, p110α and p-p85 were significantly down-regulated at the concentration of 50 nM.

To determine whether CS-6 also inhibited Hsp90 expression in other NSCLC cells, we further treated H1299, H322 and H460 cells with CS-6 at different doses, and found that CS-6 also considerably suppressed Hsp90 protein expression in H1299, H322 and H460 cells (Figure 5C).

### Identification of potential protein targets for CS-6

**Figure 6:**
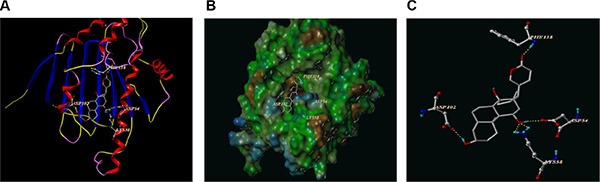
The best ranked pose of CS-6 in the ATP binding site of Hsp90 generated with docking (**A**) Interactions of CS-6 and Hsp90 are delineated by ribbon structure, Hydrogen bonds are displayed as yellow dashed lines, and the participating amino acid residues are marked. (**B**) MOLCAD representation the molecular lipophilic potential surface upon the bioactive pose of CS-6 in the ATP binding site of Hsp90. The blue denotes the hydrophilic, brown for the lipophilic and green corresponds to the neutral moiety. (**C**) The interaction between the residues of Hsp90 and CS-6 were displayed directly without the whole protein in cartoon.

Since treatment of cultured cells with known Hsp90 inhibitors depletes the Hsp90-dependent proteins in a concentration-dependent fashion [18, 19], our results also showed CS-6 could affect Hsp90 “client” proteins levels. Therefore, we hypothesized that CS-6 might bind to Hsp90 and subsequently inhibited its function. To test this hypothesis, computer molecular modeling assay was conducted to simulate the interactions between CS-6 and Hsp90. Molecular docking studies predicted that CS-6 would bind at the ATP binding site of Hsp90. As shown in Figure 6A, CS-6 forms five hydrogen bonds with the ATP binding pocket of the Hsp90 nucleoside domain. The CO motif at the lacton ring of CS-6 forms a hydrogen bond with the backbone NH of PHE138. The OH group at the C14 position forms strong hydrogen bonds with the backbone at ASP54 and LYS 58 simultaneously. It worth note that two hydrogen bonds were formed between the LYS 58 and the O of 11 hydroxyl group (Figure 6C). Moreover, the OH at the C-2 accepts a hydrogen bond with the CO residue of ASP102. The result of MOLCAD surface modeling shows that the lacton ring of CS-6 extends into the deep hydrophobic cavity of the ATP-binding pocket of Hsp90, forming the hydrophobic interaction with PHE138. Therefore, our molecular modeling predicts that CS-6 binds in the ATP-binding domain of Hsp90 with interactions (Figure 6B).

In addition, as a biochemical hallmark of Hsp90 inhibition, elevation in Hsp70 levels is indicative of ATPase activity Hsp90 ATPase activity inhibition [20]. We also detected the expression of Hsp70 after CS-6 treatment, and found that expression of Hsp70 elevated in a dose-dependent manner (Figure 4).

### CS-6 suppressed the expression of Hsp90 in nude mice tumor tissue

**Figure 7:**
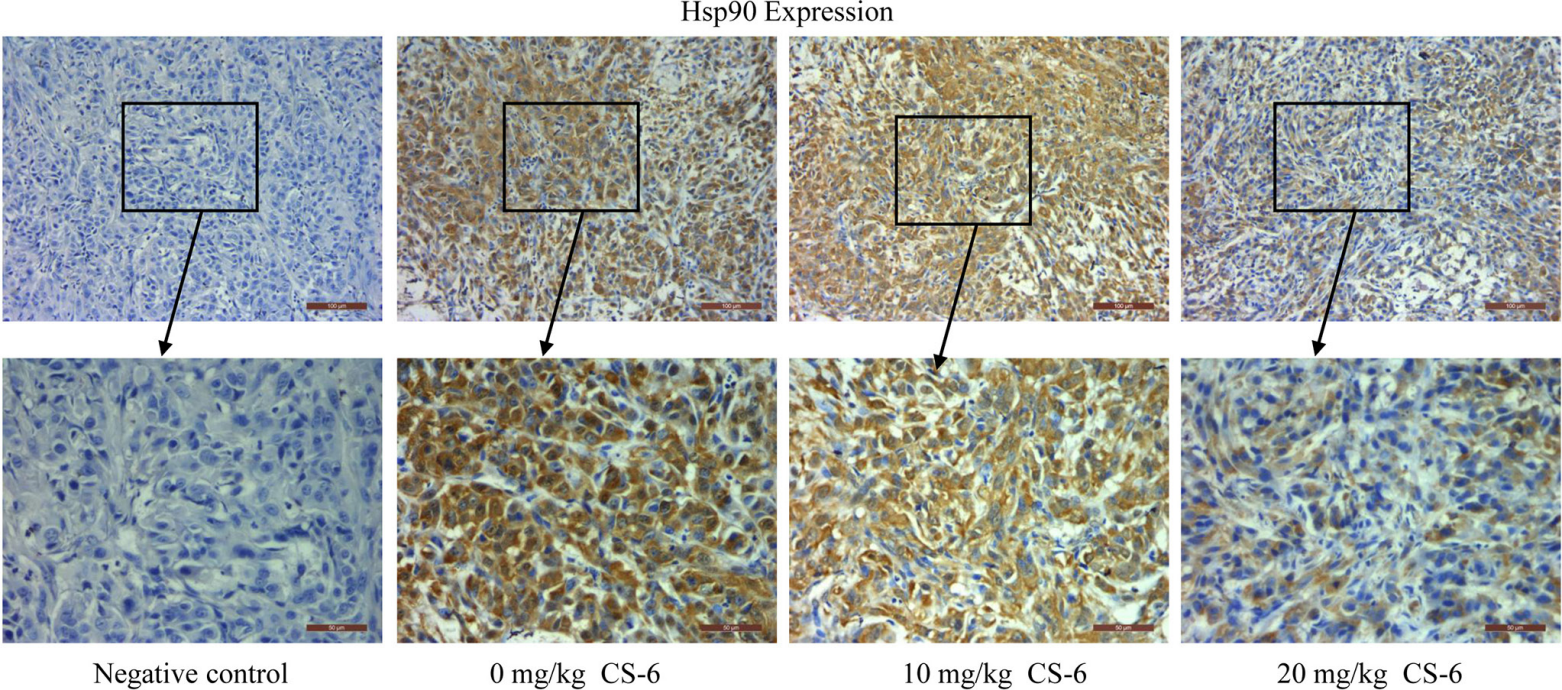
Immunohistochemical analysis of Hsp90 expression in tumor samples Neutral formalin fixed tumor samples were prepared from animals and analyzed by immunohistochemical staining with rabbit anti-rabbit second antibody using the Vectastain Elite ABC kit, and examined under a microscope.

Based on the results of *in vitro* studies, the potential of CS-6 as a novel Hsp90 inhibitor were further explored in mice with human lung cancer xenografts. Our previous experiment found that CS-6 could significantly decrease tumor volume and weight compared the control group without obvious toxic effects in mice [9]. Moreover, the immunohistochemical staining assay was used to determine the expression of Hsp90 in tumor issues. The expression levels of Hsp90 were significantly decreased with CS-6 treatment *in vivo*, as compared with the vehicle group (Figure 7); again the results of immunohistochemistry confirmed the reliability of the proteomic analysis. These results suggested that CS-6 could inhibit Hsp90 expression *in vivo*, and then inhibit xenografted human lung cancer cell's growth.

## DISCUSSION

Despite the latest technological and clinical advances, the survival rate of lung cancer has remained unchanged. Newer mechanistically driven approaches for treatment of lung cancer are in great demand. A number of scientific studies have suggested that, gamabufotalin (CS-6), the major derivative of bufadienolides, has great promise in the therapy of lung cancer.

In our present study, CS-6 was found to effectively inhibit NSCLC cells growth and enhance apoptosis induction dose-dependently with IC50 of only 50 nM. In addition, the results of the flow cytometry assay indicated that CS-6 might induce cell cycle arrest at G2/M phase and trigger apoptosis in A549 cells. Furthermore, a comprehensive proteomics was employed to characterize the proteome of resting and CS-6 treated human non-small-cell lung cancer (NSCLC) A549 cells, which might provide potential anti-cancer mechanism or the therapeutic targets of CS-6 in lung cancer. The proteomics analysis result revealed the differential regulation of the 38 proteins involved in binding, catalytic activity, structural molecule activity and enzyme regulator activity. It is noteworthy that the new and most interesting finding in our proteomic and bioinformational analysis result is the possible involvement of Hsp90 function in the effect of CS-6. Besides, the results of network construction also suggested the central role of Hsp90 in all proteins identified in the proteomics study.

Hsp90 is responsible for managing protein folding and quality control in the crowded environment of the eukaryotic cells, which facilitates the maturation and stabilization of over 200 oncogenic client proteins crucial for oncogenesis [21, 22]. Hsp90 client protein Akt, which promotes transcription of pro-survival genes, triggers NF-κB activation by regulating κB kinase (IKK) [23, 24]. Our early study had shown that CS-6 also decreased the expression levels of p-IκB-α and p-IKKβ, and inhibited translocation of the NF-κB p65/p50 proteins from cell cytoplasm to nucleus compared with control group [9]. These results indicated that CS-6 might be a potential Hsp90 inhibitor.

Cellular senescence, a permanent and irreversible process, which could induce cell growth arrest without massive cell death [25, 26]. Recently, the induction of cellular senescence, especially upon Hsp90 inhibition [27–29], has been proposed as a novel approach to enhance efficacy of cancer therapy with less severe side effects than cytotoxic therapies and high dose radiation. In the present study, CS-6 was found to down regulate several cell growth and cellular senescence associated Hsp90 client oncoproteins, including CKD4, Akt, hTERT, HIF-1α, VEGF, HER2 and VEGF. Also, we reported the correlation between CS-6 and the down regulation of Cyclin D1, p110α and p-p85. These findings suggested that CS-6 inhibit cell growth and induce cellular senescence in A549 cells by down regulating cell growth and cellular senescence associated Hsp90 client proteins.

Our previous work demonstrated that CS-6 targeted IKKβ/NF-κB, one member of Hsp90 client protein, to prohibit lung cancer growth. In the present study, we proposed that the anti-cancer activity of CS-6 is mediated, at least in part, by its ability to inhibit Hsp90. From above all, we believed that CS-6 showed double suppression of NF-kB activation signal pathway though inhibiting of Hsp90 and IKKβ activation.

Moreover, computational molecular docking implied that CS-6 occupied the deep hydrophobic pocket of Hsp90 with the ATP-binding sites (ASP54, LYS 58, ASP102, and PHE138), which suggested that CS-6 might block the nucleotide recognition domain binding with ATP, as a reversible inhibitor.

Based on these results, we predicted that CS-6 might have effect on Hsp90 function. To certify the prediction, the effect of CS-6 on cellular Hsp90 activity was checked by immunohistochemical staining assay. The expression levels of Hsp90 were found to be significantly decreased after CS-6 treatment *in vivo*, suggesting that CS-6 could inhibit cellular Hsp90 activity.

All these results suggest that targeting on multiple oncogenic pathways by CS-6, through inhibition of Hsp90, may provide an exciting novel approach in the treatment of NSCLC.

## MATERIALS AND METHODS

### Chemicals and reagents

Gamabufotalin (CS-6) was isolated from *ChanSu* by Dr. Xiaochi Ma (Dalian Medical University, Liaoning, China) [9], which was secreted from the postauricular and skin glands of Bufo bufo gargarizans Cantor. The crude materials of *ChanSu* were purchased from Qingdao (Shandong Province, China). In the experiments, the CS-6 was stocked in dimethyl sulphoxide (DMSO) and kept at – 20°C. CS-6 was diluted into desired concentration, which was stable in the dilution with DMSO less than 0.1%. RPMI 1640 and fetal bovine serum (FBS) were obtained from HyClone Laboratories (HyClone Laboratories Inc.). Dimethyl-labeled agents, CH_2_O, CD_2_O, NaCNBH_3_ were obtained from Sigma-Aldrich Company (St. Louis, MO).

All other agents were purchased from Sigma Chemical Co. (St. Louis, MO) unless otherwise specified. The water used in the experiments was thrice-distilled; all other materials were of analytical reagent grade.

### Antibodies

The primary antibodies for Hsp90 (11405-1-AP), Hsp70 (25405-1-AP), ARFIP1 (17726-1-AP), PNN (18266-1-AP), NAP1L1 (14898-1-AP), ECSH1 (11305-1-AP), PTGES3 (15216-1-AP), VEGF (19003-1-AP), GAPDH (60004-1-Ig), β-actin (20536-1-AP) and all the secondary antibodies were obtained from Proteintech Group (Proteintech Group, Inc., USA). The primary antibodies for HIF-1α (sc-10790) and hTERT (sc-7212) were obtained from Santa Cruz Biotechnology (Santa Cruz, CA, USA). The primary antibodies for Akt (4691), p-Akt (4060), p110α (4249), and p-p85 (4228) were obtained from Cell Signaling Technology (Cell Signaling Technology Inc., USA).

### Cell culture

Human NSCLC A549 cell line was from ATCC (Manassas, VA). Cells were maintained in RPMI 1640 medium supplemented with 10% FBS, 100 units/ml penicillin and 100 μg/ml streptomycin. The cells were maintained at 37°C with 5% CO_2_ in a humidified atmosphere.

### Cell viability assay and morphological changes

Cell viability was determined by 5-diphenyltetrazolium bromide (MTT) assay (Sigma). Briefly, A549 cells (4 × 10^3^ cells) were seeded in 96-well plates in normal growth medium and allowed to adhere for overnight. Then the medium was replaced with normal medium containing various amounts of CS-6 (0, 5, 10, 50, 100 and 500 nM) for 24, 36 and 48 h. After that, 15 μL MTT solution was added, and the growths of cells were measured after 4 h of incubation. To examine the effect of CS-6 on cell morphology, the cell images were captured with a Leica DM 14000B microscope after treated with appropriate CS-6. All experiments were performed in triplicate.

### Cell cycle analysis

Cell cycle analysis was conducted by FACS Accuri C6 (Genetimes Technology Inc.) to evaluate the effects of CS-6 on cell cycle arrest. Briefly adherent and detached cells were harvested with trypsin, washed with PBS three times, and then fixed in ice-cold 70% ethanol at 4°C for 4 h. After centrifugation at 1000 rpm for 5 min, cells were resuspended in propidium iodide stain buffer (0.2% Triton X-100, 100 μg/mL DNase-free RNase A, and 50 μg/mL propidium iodide in PBS) for 30 min in the dark. Flow cytometric analysis was conducted using FACS Accuri C6 (Genetimes Technology Inc.).

### AO/EB staining

Acridine orange/ethidium bromide (AO/EB) staining is used to visualize nuclear changes and apoptotic body formation. The A549 cells (2 × 10^5^ cells/mL) were seeded in 6-well plates for 24 h and treated with various concentrations of CS-6 (0, 10 and 50 μM) for 36 h. After incubation, the cells were washed with PBS. Then, the mixture solution containing same volume of AO and EB (diluted in PBS for 100 μg/ml) was put onto the cells. The images of the cells were observed using a Leica DM 14000B fluorescence microscope fitted with digital camera.

### Protein preparation and tryptic digestion

A549 cells were exposed to 50 nM CS-6 for 36 h, and then harvested for protein extraction on ice. The cell pellets were dissolved in a cell lysis buffer (8 M urea) plus 1% (v/v) protease inhibitor cocktail set. The suspension was homogenized on ice for 1 min, ultrasonicated for 30 s and centrifuged at 25000 g for 30 min at 4°C, and the supernatants were harvested and frozen at – 80°C. Protein concentrations were determined using a BCA protein assay kit according to the manufacturer's instructions. Proteins were reduced with 10 mM dithiothreitol for 30 min at 56°C, then alkylated with 20 mM iodoacetamide for 1 h at room temperature in the dark. Cell lysates were diluted and digested with a trypsin to protein ratio of 1:25 (W/W) at 37°C overnight. The resulting peptide solutions were acidified with 1% FA, and desalted on a C18 trap column. Eluted peptides were lyophilized to complete dryness, and stored at – 80°C until needed.

### Stable isotope dimethyl labeling

Stable isotope dimethyl labeling was performed according to the reported protocol [12] with the appropriate improvements. Briefly, the samples were labeled by light (0.2% CH_2_O and 30 mM NaBH_3_CN) and heavy (0.2% CD_2_O and 30 mM NaBH_3_CN) dimethylation reagents, respectively. After keeping the reaction solution in 25°C for 1 h, 2 mL of 10% (vol/vol) ammonia and 5 mL of 10% (vol/vol) formic acid in water were successively added to quench the reaction. The isotopically labeled peptides were mixed together, desalted by a C18 solid-phase extraction column, lyophilized to powder and re-dissolved in 0.1% FA in H_2_O for the following LC-MS/MS analysis.

### NanoLC−MS/MS analysis

The peptide samples were analyzed by nano-RPLC-ESI-MS/MS with an LTQ-OrbitrapElite mass spectrometer (Thermo Fisher Scientific, San Jose, CA, USA) equipped with a Dionex ultimate 3000 liquid chromatography and an ESI probe Ion Max Source with a nanospray kit. The spectrometer was controlled by Xcalibur software version 2.2 (Thermo Fisher, Waltham, MA, USA). The peptides were separated on a C18 capillary column (30 cm, 75 μm i.d./375 μm o.d.) packed with C18 silica particles (5 μm, 100 Å) with a 145 min gradient from 10 to 40% buffer B (98% ACN/0.1% FA) and analyzed on the mass spectrometer. Mass spectra were acquired in a data-dependent mode. MS1 spectra were measured at a resolution of 6 × 10^4^ and the top 10 most abundant ions with an isolation window of 2 m/z were selected for sequencing and fragmented in the data-dependent CID mode with a normalized collision energy of 35%, activation Q of 0.25, activation time of 10 ms, and one microscan. The sample was analyzed in triplicate.

### Data processing

Data analysis was accomplished by using MaxQuant software (http://maxquant.org/, version 1.3.0.3) against IPI human database (v3.80). Peptides were searched using the following parameters: fully tryptic cleavage constraints; up to two internal cleavage sites allowed for tryptic digestion; carbamidomethylation as a fixed modification; oxidation of methionine and protein N-terminal acetylation as variable modifications; dimethyl (+ 28.0313 Da) and dimethyl (+ 32.0564) N-termini and K set as light/heavy labels for quantification. The peptide mass tolerance was set at 20 ppm and MS/MS tolerance was set at 0.5 Da. Protein and peptides FDRs were 1%. The rest of the parameters follow the default settings of MaxQuant software.

### Bioinformatics analysis

Gene Ontology annotations including cellular component, biological process, and molecular function were performed with Blast2GO program (Version 2.7.0) [30–32]. A global protein-protein interaction network was generated using Cytoscape (Version 3.1) [33, 34].

### Western blot analysis

A549 cells were treated with various concentrations of CS-6. Total cell proteins were prepared using mammalin protein extraction reagent (purchased from Beijing CoWin Bioscience Co., Ltd.). The concentration of proteins was determined using a BCA protein assay kit. Cell lysate proteins (40 μg) were separated by electrophoresis on a 7.5–12% sodium dodecyl sulfate-polyacrylamide minigels (SDS-PAGE) gels and then electrophoretically transferred to PVDF membranes. After that, the membranes were incubated with 5% dehydrated skim milk for 2 h at room temperature. Western blots were probed with the specific antibodies. Proteins were detected by enhanced chemiluminescence system according to the manufacturer's instructions. Similar experiments were performed at least three times.

### Molecular modeling

The molecular docking studies were performed to explore the potential binding mode between CS-6 and Hsp90 protein complex. CS-6 was optimized using the semi-empirical PM3 method with the Polak-Ribie're conjugate gradient algorithm with an RMS gradient of 0.01kcal mol^−1^ Å^−1^ as convergence criterion. The optimized structure of CS-6 was docked into the active site of Hsp90 with ligand (PDB Code: 3QDD). The crystallographic ligand was extracted from the active site, and the Surflex-Dock program was used for the docking calculations with default parameters. MOLCAD surfaces were generated for visualizing the binding mode of the docked protein-ligand complexes

### Animal studies

As reported in our previous work [9], all animals were maintained, and animal experiments were done in SPF Laboratory Animal Center at Dalian medical university. A549 cells (2 × 10^6^ in 100 μL PBS) were injected subcutaneously near the axillary fossa of female nu/nu mice using a 27-gauge needle. The tumor cell-inoculated mice were randomly divided into three groups: group A was treated with PBS; group B with 5 mg/kg CS-6; group C with 10 mg/kg CS-6 by intraperitoneal injection every day. After treated with CS-6 for two weeks, all experimental mice were killed with ether anesthesia. To determine Hsp90 expression in neoplastic tissues, the tumors were harvested and freshly fixed in 10% neutral formalin and desiccated and embedded paraffin. 4 μm sections were stained with specific Hsp90 antibody (1:100). The images were examined under a Leica DM 14000B fluorescence microscope equipped with a digital camera.

### Statistical analysis

All experiments were repeated at least three times. Data are represented as mean ± standard deviation (SD). Analysis of variance and Student's *t*-test were used to compare the values of the test and control samples *in vitro* and *in vivo*. *P* < 0.05 was considered to be a statistically significant difference. SPSS 17.0 software was used for all statistical analysis.

## CONCLUSIONS

Our study demonstrated the *in vitro* and *in vivo* antitumor effect of a novel Hsp90 inhibitor, CS-6, on the NSCLC cell line A549. To the best of our knowledge, it might be the first time to report the treatment of CS-6 on Hsp90 expression and to demonstrate the underlying mechanisms both *in vitro* and *in vivo*. CS-6 inhibited cell growth, cell migration, tumor sphere formation and induced cellular senescence in A549. By using a proteomics technique and bioinformatics analysis, 38 target-related proteins and the regulated network of CS-6 were found. The ability of CS-6 to target multiple NSCLC oncoproteins, make it a potent antitumor agent in treatment of NSCLC. Together with the tumor suppressive effect of CS-6 in nude mice tumorigenicity assay, this study provided preclinical evidence of using CS-6 as a new therapeutic agent in treatment of NSCLC. The results of the present study shed light on the anticancer mechanism of CS-6 from a molecular perspective. Besides, understanding of the cytotoxicity mechanism of CS-6 will be helpful to the study the use of likely promising bufadienolides. Furthermore we are continuing to find new bufadienolides from toad venom and other herbs. It is possible that we can obtain promising bufadienolides for cancer therapy either by isolating from herbs or by modifying natural bufadienolides.
